# The perceived role of ward-based primary healthcare outreach teams in rural KwaZulu-Natal, South Africa

**DOI:** 10.4102/phcfm.v9i1.1388

**Published:** 2017-05-29

**Authors:** Landiwe S. Khuzwayo, Mosa Moshabela

**Affiliations:** 1 School of Nursing and Public Health, University of KwaZulu-Natal, South Africa; 2 Department of Rural Health, University of KwaZulu-Natal, South Africa

## Abstract

**Background:**

The aim of ward-based outreach teams (WBOTs) is to improve access to primary healthcare (PHC) services including health promotion and disease prevention in South Africa. Limited information is available in South Africa on user perceptions of services provided by WBOTs in rural households.

**Aim:**

The study aimed to explore community awareness and perception of WBOTs, as well people’s motivation to engage and use WBOT services.

**Setting:**

The study was conducted between July and September 2015 in iLembe district, KwaZulu-Natal.

**Methods:**

This was exploratory-descriptive qualitative research. Purposive sampling technique was used in this study. A total of 16 key informant interviews and 4 focus group discussions were conducted. The voice recordings were transcribed in isiZulu and translated into English.

**Results:**

Four themes emerged from the data analysis, namely bringing services closer, organising services, expanding services and forming bridges. Respondents demonstrated insightful knowledge and understanding of services provided by WBOTs. They expressed an appreciation of the way WBOT services brought healthcare closer to people and serve to bridge the gap between the community and local healthcare facilities. Respondents identified unclear WBOT work schedules and the failure to carry medication other than vitamin A as the main challenges. However, WBOTs did deliver medication for controlled chronic patients in their households.

**Conclusion:**

The study suggests that WBOTs provide a commendable service, but need to expand their service package to further increase access to PHC services and cater for community health needs.

## Introduction

More than 30 years after the 1978 Declaration of Alma Alta,^1,2^ and in line with the World Health Organization’s (WHO) advocacy for the revitalisation of primary healthcare (PHC),^1^ the South African Government developed the 10-Point Plan to transform the healthcare system of the country.^2^ Central to this transformation is the strengthening of PHC as the backbone of health service delivery,^2^ and the National Health Insurance (NHI) as a long-term goal for providing equitable access and universal coverage for a defined package of healthcare.^3^

PHC re-engineering is one of the key pillars of the NHI and consists of a four-stream approach which includes contracting private health practitioners at non-specialist level, district-based clinical specialist teams, strengthening school health services and ward-based PHC outreach teams for each electoral ward with an initial focus on improving maternal and child health.^4^ The core principles of PHC re-engineering are to attain a population-orientated view to healthcare, including prevention, promotion and good quality; focus on health outcomes, aimed at reducing mortality and morbidity from the major causes of ill-health; develop integrated, efficient and well-supported PHC teams, guided by and accountable to communities; establish a well-functioning District Health System (DHS); and to pay closer attention to those factors outside of the health sector that impact health, namely the social determinants of health.^2^

Theoretically, PHC ward-based outreach teams (WBOTs) are composed of a professional nurse, environmental health and health promotion practitioners, and four to five community health workers. Each ward is expected to have at least one team that will serve a population of about 7600.^5^ The role of WBOTs is to promote child, adolescent and women’s health; provide basic preventative care and health promotion and education; identify people at risk; support adherence in chronic care; offer home-based care; assist in early detection and intervention of health problems and illness; offer basic first aid and emergency interventions; and help integrate care at the community level.^3,4,6^ These services are regarded as primary care services as they offer the first point of entry to the health system, provided by a team of health workers, and are focused towards disease prevention, individuals and families.^7^

The main aim of the WBOTs is to improve access to PHC in South Africa, as they provide a range of PHC services including health promotion and disease prevention.^8^ There is little research on user perception of services provided by WBOTs, especially in rural communities. The intention behind the introduction of WBOTs is to meet people’s service needs in communities; how these services are perceived matter as they may not meet local expectations. Knowing users’ perceptions is important as it allows service providers to offer services that respond to users’ needs.

User perception studies are important as they provide an opportunity for users to express themselves, and to encourage their participation in the planning process of services to be provided. User perception studies also allow service providers to evaluate the services being delivered, with the potential to improve routine health services.^9^ The study aimed to explore awareness and perceptions of WBOTs, as well as people’s motivation to engage and use services provided by WBOTs in community. The perceptions of clients and users should help policy and decision makers to implement programmes tailored to patients’ needs as perceived by patients and service providers.^10^

This study was conducted in iLembe district in KwaZulu-Natal province (South Africa). The district has a total population of 606 809.^11^ The district has three WBOTs that are linked to Mpumelelo, Isithebe and Groutville clinics. Research was undertaken in the rural inland areas that fall under traditional authorities.

## Research methods

This study is a qualitative descriptive enquiry. Four focus group discussions (FGDs) and 16 key informant in-depth interviews (KII) were conducted with community leaders and members. The minimum and maximum number of participants for FGDs was 4 and 14, respectively. A community leader was defined as a person who is perceived to represent a community, and household members were defined as representatives of households who were most knowledgeable about the household’s health status and health history over the preceding 12 months. Participants were purposively selected based on their knowledge and experience of WBOTs and the WBOT services offered in the households. The community leaders assisted with the recruitment of participants.

Data collection was carried out between July 2015 and September 2015. Key informant interviews and FGDs were conducted in isiZulu (the local language) by the researcher and a trained research assistant, both of whom are native speakers. In-depth interviews were conducted in people’s homes while FGDs were conducted at community halls. Topic guides were used for both the in-depth and focus group interviews. All interviews and FGDs were audio-recorded.

Data were transcribed and translated. They were coded using NVivo 10 and analysed using a conceptual framework developed from a study of access to medical care adapted from Aday and Andersen.^12^ Using inductive categories, broad themes were identified and power codes and proof codes from narratives of key informants and focus group participants were generated. Through an iterative process, similar proof and power codes were grouped under themes,^13^ while themes were refined and clearly charted. This article uses data from the themes that describe the services provided by the WBOTs.

### Ethical consideration

The study was approved by the Biomedical Research Ethics Committee of the University of KwaZulu-Natal (BE209/15).

## Results

A total of 44 participants, 16 KII and 28 participants for FGDs, were interviewed: 3 men and 41 women. There were one ward councillor and two community development workers among the participants. The remainder were community members. The age range of the participants was between 31 and 62 years, with an average age of 42 years.

### Themes and subthemes

Analysis of the data yielded four main themes: (1) bringing services, (2) organising services, (3) forming bridges and (4) expanding services. The themes are presented in Table 1, and further explored below.

**TABLE 1:** Themes and subthemes.

Theme	Subtheme
Bringing services	Provision of health education Chronic medicine carrier in certain circumstances
Organising services	Working schedule Adequate time spent per visit
Forming bridges	Reduced costs associated with seeking healthcare services Referral of other community members to WBOT
Expanding services	Referral of patients to higher level of care Current package of care

*Source*: Authors’ own work

### Bringing services

Participants said that WBOTs provided both educational and practical services. In terms of health education, topics covered included maternal health, especially the importance of attending ante- and postnatal visits, child health, disease prevention, chronic disease management and referral to clinics. WBOTs provided direct basic services including maternal health checks, and vitamin A and growth monitoring in children. When professional nurses were present they also offered vaccination services.

‘They also visit pregnant women, and check if they have been to the clinic for antenatal care [*ANC*] services and they would come back to check on the women after they have given birth and also to see if the mother has taken the child to the clinic for vaccination.’ (FGD, Isithebe, female, community member)

Referrals to clinics by the teams were regarded as an important aspect of bringing services closer to the communities. Respondents said that they would not have gone to the clinic had they not been referred by the WBOT, for example for deworming of children, or continuation of care for complicated cases. Respondents also said that WBOTs carried basic medical supplies and delivered chronic medication for stable chronic patients. Some participants thought this service should be expanded because of the need for more medical supplies to be brought closer to the community.

‘They do carry pain tablets, and chronic medication for chronic patients if they know what condition they have maybe arthritis or BP, they would bring them.’ (KII, Groutville, female, community member)

### Organising services

Respondents report a lack of communication about visit scheduling. Most said that they did not know when the team would come and that they did not appear to follow a clear schedule, they just saw them when they appeared. WBOT visits depended on the problems each household was having, and were handled on a case-by-case basis.

Some, however, said they could discern a work schedule. They said that on certain days WBOTs would come to conduct follow-up on previously seen cases, and on other days they would deliver chronic medication for stable patients, administer vitamin A and also provide health education.

‘Their visit depends on the household, in terms of how much problems do the household have, if they need to do follow up visits, they would then come back for follow-up to monitor those cases.’ (FGD, Isithebe, female, community member)

WBOTs also specifically visited the elderly people on certain days, while on other days they focused on child health-related issues.

‘Yes they have days because some other time they will come to the old people, and on other days they will do the children.’ (KII, Groutville, female, community member)

In terms of time spent per visit, most respondents mentioned that the duration of the visit depended on the nature or the purpose of their visit to the particular household and that generally they spent at least 30 minutes at their homes.

‘The time they spend depends on the nature of your condition.’ (KII, Sadloko, female, community member)

### Forming bridges

Respondents said that WBOTs play an important role in reducing out-of-pocket expenses and improving their access to services. They said that the areas serviced by WBOTs are vast and clinics are far from communities. They further said that in the past they were unable to go to the clinic to collect medicine when they did not have money for transport. However, since the introduction of WBOTs, they reported that they no longer needed to go as often, and that they went only when they were referred by the WBOT or when it was necessary.

‘There is no need to catch a taxi to the clinic, unless they are the ones that are sending you to the clinic.’ (KII, Groutville, female, community member)

The presence of WBOTs in communities brought some sense of relief to people, as they presented them with an opportunity to learn and ask about things they did not know or understand before the introduction of the teams. Respondents actively encouraged fellow community members to use their services. As one put it:

‘Yes, I would advise them. My other brother passed on in November. He was looking for assistance and I told him to contact them, because people are not the same, there is a person that makes you understand things and put your mind at ease.’ (KII, Isithebe, female, community member)

### Expanding services

The current package of care for WBOTs was regarded as insufficient for community needs. With the exception of pre-packed prescriptions for known stable chronic patients, WBOTs did not supply medications. Participants felt that some of the basic PHC services, such as child vaccinations and deworming, should be shifted to the WBOTs to reduce referrals to the clinic.

‘May they please carry deworming medication as well, because it is added time going to the clinic for deworming only and not for vaccination, you end up overcrowding the clinic.’ (FGD, Isithebe, male, community member)

Referrals were perceived to occur largely because WBOTs offered limited services and could not handle complicated cases.

‘Because they don’t carry lots of things, they make a referral letter to the clinic; I think that is a nice thing about them.’ (FGD, Groutville, female, community member)

While WBOTs seem to provide patients with referral letters some report that, at times, these were not given. Respondents also gave WBOTs feedback on referrals and their facility visits. Among study participants, there were some who had not had contact with WBOTs, mainly because they were close to a health facility. Some also reported difficulty accessing WBOT services when they needed them, either because there were too few teams to service the need and they came only after long intervals or because the healthcare professionals attached to them were unavailable when needed.

‘Yes there are problems as we are saying, there is only one team that we normally see, it will happen that you need the professional nurse, and when you phone, you will find that he is attending a workshop or maybe he is in a meeting and he ends up not coming when you need him. So those are our problems.’ (FGD, Isithebe, female, community member)

## Discussion

The study reveals that WBOTs are regarded as a valuable resource that provides people with much needed access to healthcare in remote and rural communities. In particular, the functions and activities of the WBOTs were regarded as bringing services closer to people, mostly through the provision of health education, by delivering chronic medication for clinically stable patients and by making referrals to clinics and follow-ups after clinic visits. Clinic access was especially effective when people were provided with letters of referral. Respondents in this study recognised the importance of these decentralised services, pointing out that people’s access to facilities was greatly influenced by the time and money involved in reaching facilities. Services provided by WBOTs showed potential to help reduce unnecessary clinic visits as well as related costs of transport to healthcare facilities. The introduction of WBOTs has lightened the burden associated with seeking healthcare as they now incur less costs, and benefit from availability of community-based healthcare and managed referral pathways. Research on HIV or AIDS and tuberculosis (TB) treatment adherence has shown that the leading cause of loss to follow-up and treatment default among antiretroviral and TB patients is because of out-of-pocket expenses, particularly patient inability to pay for the transport costs for clinic visits.^14^ Conversely, a study in rural South Africa found that down referral to community-based services resulted in fewer access barriers, saved time and money on clinic days and showed improved antiretroviral therapy (ART) adherence,^15^ although there were concerns about compromised quality of care.^15^

The study found that WBOTs services formed a bridge between households and PHC facilities, and this bodes well for WBOTs since their main goal is to improve access to PHC services. Community health workers, who are also members of the WBOTs, have been shown to bridge the gap in service delivery, help strengthen the link between the community and healthcare system,^14^ ensure better patient retention and treatment adherence, and therefore contribute to improvement in prioritised health outcomes.^6,16^ Similar to previous studies, the role of WBOTs in linking communities with the health system is not clearly defined with regard to ensuring that community members enter and remain in the healthcare system, and this gap needs to be addressed.

While WBOTs appear to improve people’s understanding of and ability to navigate health services, findings from this study also suggest that community members would benefit from a more expanded and reliable system of outreach teams. Reliability of WBOTs could be enhanced by explicit and consistent scheduling of visits, a more accessible and responsive system in cases where the need to contact team members arise, and the expansion of the package of services provided. WBOTs need to provide a comprehensive primary care package of services in communities, as done in other developing countries that offer similar services.^1,6^ The responsibilities of Family Health Teams (FHTs) in Brazil and India include patient-centred care, as well as timely and appropriate access to extended hours of care.^17^ While the literature on does not seem to describe their work schedules, our research suggests that WBOTs need to have work schedules that are consistently applied and known to the communities they serve.

The current package of care provided by WBOTs does not appear to meet people’s needs. This may be a result of perceptions if the package of services is not well-discussed or conveyed and can lead to client dissatisfaction and strained relationships.^16^ Findings from this study suggest that it is also a fact, the current package of care needs to be expanded, perhaps by further decentralising some of the basic PHC services to WBOTs in order to cater for community needs and minimise referrals to the clinic. Service packages delivered by community health workers are known to be crafted individually for each household, and needs for each client is central to the process of negotiating care.^16^ The WBOTs visits to the household depend on the family needs, and the WBOTs will conduct follow-up visits on previously seen cases. These household follow-ups are similar to what the FHT does, where they are responsible for a permanent and systematic follow-up of a given number of families residing in a circumscribed area, and for establishing ties of commitment and shared responsibility.^6^

## Conclusion

This study shows that WBOTs are a welcome extension of PHC into communities, especially for people who live in remote and rural areas. In analysing perceptions of WBOTs, people understand and value the services WBOTs are providing, as they have increased access to their healthcare and reduced out-of-pocket expenses. In this way, they make an important contribution to national government efforts to provide healthcare for all. The reach of WBOTs and the current package of service provided by them are limited, however, and are a cause of some dissatisfaction that needs to be addressed.

### Recommendations

The recommendations below are presented based on the finding and conclusion of the study:

- A working schedule for the WBOTs should be developed and shared with the community.
- The current package of care should be expanded to include performance of procedures such as child vaccination, deworming and handling of complicated cases.
- Decentralising basic PHC services to WBOTs in order to decongest the clinics.
- Increasing the number of teams per ward should be advocated for in order for the team to visit households frequently.

### Limitations

The study is a qualitative study based on perceived experiences of purposively selected individuals in a defined community in rural KwaZulu-Natal. It is limited in scope and scale, providing insights into a particular aspect of primary care re-engineering at one point in time. The findings are indicative and not necessarily generalisable.
